# Maternal and Perinatal Outcomes in Pregnant Women with Heart Disease: A Case—Control Study

**DOI:** 10.3390/jcm13175084

**Published:** 2024-08-27

**Authors:** Irene Aracil Moreno, Raquel Prieto-Arévalo, Virginia Ortega-Abad, Virginia Martín-Manzano, Laura Pérez-Burrel, Andrea Fraile-López, Carolina Devesa-Cordero, Fátima Yllana-Pérez, Miguel A. Ortega, Juan A. De León-Luis

**Affiliations:** 1 Department of Public and Maternal and Child Health, School of Medicine, Complutense University of Madrid, 28040 Madrid, Spain; irene.aracil@salud.madrid.org (I.A.M.); virginia.ortega@salud.madrid.org (V.O.-A.); lpburrel@salud.madrid.org (L.P.-B.); andrea.fraile@salud.madrid.org (A.F.-L.); lourdesfatima.yllana@salud.madrid.org (F.Y.-P.); jaleon@ucm.es (J.A.D.L.-L.); 2 Department of Obstetrics and Gynecology, University Hospital Gregorio Marañón, 28009 Madrid, Spain; 3 Health Research Institute Gregorio Marañón, 28009 Madrid, Spain; raquel.prieto@salud.madrid.org (R.P.-A.); virginia.martinmanzano@salud.madrid.org (V.M.-M.); carolina.devesa@salud.madrid.org (C.D.-C.); 4 Department of Cardiology, Hospital General Universitario Gregorio Marañón, Facultad de Medicina, Universidad Complutense de Madrid, Instituto de Investigación Sanitaria Gregorio Marañón, 28007 Madrid, Spain; 5 Centro de Investigación Biomédica en Red Enfermedades Cardiovasculares (CIBERCV), 28029 Madrid, Spain; 6 Department of Medicine and Medical Specialities, CIBEREHD, Faculty of Medicine and Health Sciences, University of Alcalá, 28801 Alcala de Henares, Spain; 7 Ramón y Cajal Institute of Sanitary Research (IRYCIS), 28034 Madrid, Spain

**Keywords:** heart disease, delivery, parity, pregnancy

## Abstract

**Objective**: We analyzed the obstetric and cardiac characteristics and results of pregnant women with heart disease (HD) and compared their results with those of healthy controls. **Methods**: In this retrospective single-center case–control study, women with HD attended between 2010 and 2018 were matched at a 1:2 ratio (according to date of delivery, parity, and singleton or twin pregnancy) with controls without heart disease treated in the same referral center. **Results**: We identified 141 pregnant women with HD, of whom 132 reached 22 weeks of gestation and were paired with 264 healthy controls, for a total of 396 participants and 408 newborns. Most common HDs were congenital HD (53 women), arrhythmia (46), valvular HD (35), and cardiomyopathy (16), having women with more than one coexisting HD. During pregnancy or the puerperium, 19.9% of mothers experienced a major adverse cardiac event (MACE), with 5% requiring intensive care unit (ICU) admission. The rates of cesarean section were 37.1% in the case group and 18.2% in the control group, with an odds ratio (OR) of 2.66 (95% CI = 1.66–4.26, *p* < 0.001). We also found a higher use of general anesthesia, with an OR of 10.73 (95% CI = 2.32–49.75, *p* = 0.002); more prolonged hospitalizations, with an OR of 2.91 (95% CI 1.02–8.35, *p* = 0.023); and a higher incidence of low neonatal weight, with an OR of 1.96 (95% CI 1.09–3.52, *p* = 0.012). There were no differences between groups in terms of gestational age at delivery; however, we observed greater prematurity in women with HD, without reaching statistical significance. The rate of congenital heart disease among the newborns of mothers with HD was 13.2%. **Conclusions**: HD increases maternal morbidity during pregnancy and it is associated with higher rates of cesarean section and low birth weight.

## 1. Introduction

Heart disease (HD) affects 1–4% of pregnant women and is the leading cause of maternal mortality in developed countries [1,2,3]. HD includes a wide range of pathologies, including congenital heart disease, cardiomyopathy, arrhythmia, and valvular disease. In Europe and North America, the most common type of HD in pregnant women is congenital HD (75–82%), while in non-industrialized countries, rheumatic valve disease is more prevalent (56–89%) [4]. Between 2011 and 2016, cardiomyopathies and other HDs were the causes of 25% of pregnancy-related deaths in the United States, above infections, bleeding, and hypertensive disorders of pregnancy [5].

During pregnancy, physiological cardiovascular changes take place, causing an increase in blood volume and cardiac output, that could be poorly tolerated in women with structural or functional HD [6]. Women with HD have a higher risk of maternal and neonatal adverse events, including worsening of previous HD, prematurity, low birth weight, and congenital heart disease, with an increase in both maternal and perinatal mortality [7]. There exist different scales and classifications to estimate the risk of cardiovascular events during pregnancy in women with HD, being the modified World Health Organization (mWHO) risk classification the most widely used [8]. Regarding delivery in patients with cardiopathy, although the rate of cesarean section is high, there is increasing evidence that supports the safety of vaginal delivery, as long as the mother is hemodynamically stable and there are no other obstetric contraindications [1,4].

The objectives of this study were, firstly, to evaluate the maternal characteristics and the cardiovascular and obstetric events that occurred during pregnancy in women with HD. And, secondly, to compare their delivery and perinatal outcomes to those obtained in our general population, through a case–control study.

## 2. Materials and Methods

We carried out a retrospective, observational, case–control study, nested in a hospital-based cohort. This cohort is composed by all pregnant women who delivered at a single referral hospital between January 2010 and December 2018. Within this cohort, the case group included women with heart disease, previously known or diagnosed during pregnancy. Those patients in whom HD was ruled out after cardiologic evaluation and/or complementary studies, and those with lack of data due to loss of follow-up were excluded. Patients with a miscarriage or pregnancy termination before 22 weeks were excluded from the case–control analysis, however, their cardiovascular variables and characteristics were collected for the descriptive analysis of the HD group. All the cases included in the comparative study were then matched, with a 1:2 ratio, with pregnant women without HD and more than 22 complete weeks of gestation, from the same hospital cohort, that conformed the control group. The matching criteria were: number of fetuses (singleton or twin pregnancies), maternal parity, and date of delivery, with a maximum difference of 7 days. The controls were retrospectively selected from a database that includes information about the entire cohort of pregnant women treated in a single tertiary care hospital. With the intention of making the controls as representative as possible of our real population of pregnant women, their selection was blinded with respect to maternal, gestational, and neonatal outcomes, only verifying that they met the matching criteria and the absence of known cardiopathy. This study follows the STROBE recommendations [9].

Regarding pregnant women with HD, information about cardiovascular risk factors, cardiopathy characteristics, first cardiological evaluation, and obstetric–neonatal outcomes was collected. The cardiovascular risk factors and maternal comorbidities of interest were smoking, diabetes mellitus, chronic arterial hypertension, conception by in vitro fertilization (IVF), personal history of heart failure, stroke, known HD, previous heart surgery, pre-pregnancy medication, and severe complications in a previous pregnancy. The baseline cardiological characteristics and echocardiographic findings were collected from the cardiologist’s assessment reports, either the last one before pregnancy or the first one during pregnancy if there was no previous. The types of HD were categorized into congenital, cardiomyopathy, ischemic heart disease, Marfan syndrome, valvular heart disease, and arrhythmia. Palpitations without evidence of arrhythmia were excluded. In the case of coexisting types of HD within the same patient, data were collected for each one to avoid loss of information. Transthoracic echocardiogram findings included systemic ventricular ejection fraction (LVEF) less than 55%; systemic ventricle dilation, defined as an end-diastolic diameter higher than 55 millimeters (mm); subjective dilation of the sub-pulmonary ventricle; dilation of the ascending aorta (diameter higher than 35 mm); systemic ventricular outflow tract obstruction, with a peak gradient above 30 mm of mercury (mmHg); pulmonary hypertension, determined by a trans-tricuspid velocity higher than 3.4 meters per second (m/s) or 2.9 m/s if other suspicious data were associated; and the presence of moderate to severe valve regurgitation. Data on the functional class according to the New York Heart Association (NYHA) classification of heart failure and the classification of the risk of cardiovascular complications associated with pregnancy according to the modified WHO classification (mWHO) were also collected.

Adverse events that occurred during pregnancy, childbirth, or the puerperium in women with HD were categorized into cardiovascular, obstetric, or neonatal complications. Major cardiovascular complications (MACEs) recorded were heart failure, peripartum cardiomyopathy, acute lung edema, thromboembolic complications (deep vein thrombosis, pulmonary embolism, peripheral embolism, or prosthetic thrombosis), arrhythmogenic complications (defined as sustained supraventricular or ventricular arrhythmias or arrhythmias requiring treatment modification), cerebrovascular accident, cardiac arrest, aortic dissection, cardiac surgery during pregnancy, and maternal death (from the beginning of pregnancy to 42 days after delivery or pregnancy termination). Other cardiovascular variables of interest recorded were cardiological medication used during pregnancy, changes in the NYHA functional class, hospital admission for cardiovascular causes and intensive care unit (ICU) admission. With regard to obstetric complications, these included abortion, stillbirth (from 22 completed weeks of gestation), hypertensive disorders of pregnancy, threatened preterm birth, intrauterine growth restriction (IUGR), fetal macrosomia, oligohydramnios, postpartum hemorrhage (blood loss over 500 mL after a vaginal delivery or 1000 mL after a cesarean section), placental abruption and maternal hospital admission during pregnancy or the puerperium (excluding admission for usual obstetric indications). Finally, neonatal complications included: congenital heart disease, respiratory distress, hyperbilirubinemia, necrotizing enterocolitis, retinopathy, intraventricular hemorrhage, non-cardiac malformations, neonatal hospital admission, neonatal intensive care unit (NICU) admission and neonatal mortality during the first 28 days of life. Other variables of interest collected were breastfeeding and the reason for cesarean section; classified as cardiovascular, obstetric, or both, when there was no absolute indication and the decision was reached by multidisciplinary consensus between obstetricians, cardiologists, and neonatologists.

For the case–control analysis, data on the following variables were collected in both groups: maternal age, parity, singleton or twin pregnancy, gestational age (GA) at the time of delivery, onset of labor (spontaneous or induced), anesthetic technique used, type of delivery (eutocic vaginal birth, assisted vaginal birth, scheduled cesarean section, or unplanned cesarean section, when performed during labor or attempted vaginal delivery), length of maternal admission after delivery and maternal mortality. Regarding neonatal outcomes, the following data were gathered: sex of the newborn, type of neonatal resuscitation, Apgar score after one minute and after five minutes from birth, pH result in arterial blood from the umbilical cord, weight in grams, weight percentile adjusted for GA and sex, and perinatal mortality (between 22 completed weeks of gestation and 7 days after birth).

For the statistical analysis, descriptive analyses and relevant hypothesis contrasts were used based on the nature of each variable using SPSS version 21 software (IBM Co., Somers, NY, USA). The limit of statistical significance was set at *p* < 0.05.

All data for the present study were obtained by reviewing clinical records and databases designed for this purpose with the approval of the Ethics Committee for Medical Research. All the authors declare that there are no conflicts of interest.

## 3. Results

**Figure 1:**
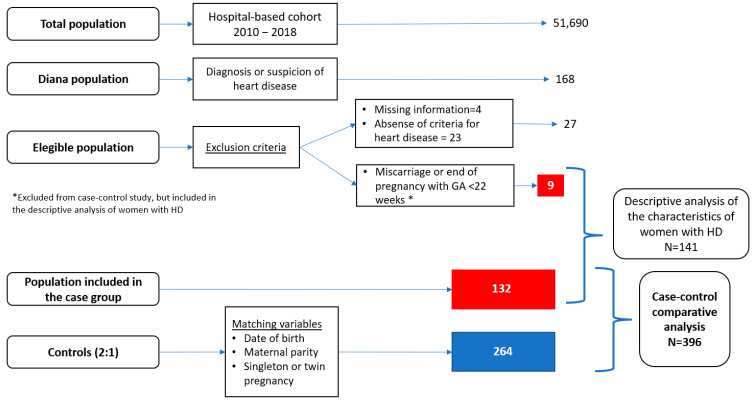
Flow chart of the study.

The flow chart of the study patients is shown in Figure 1. From 2010 to 2018, a total of 51,690 deliveries were attended in our center. During this period of time, 168 pregnant women were evaluated in the cardiology unit for known or suspected HD; 27 of them were excluded due to missing data or absence of HD, and 141 women with confirmed HD (0.27% of the cohort) were included in the study for the descriptive analysis. Among them, 9 pregnancies did not reach 22 completed weeks, due to 8 spontaneous miscarriages and one voluntary termination of pregnancy related to fetal HD. This brings us to a total of 132 women with HD and evolving pregnancy that were finally included in the case–control comparative study and matched with 264 healthy controls. Out of all, 3% of them had twin pregnancies in both groups, therefore, the case-control study included a total of 396 women and 408 newborns.

Basal cardiovascular characteristics of women with HD are shown in Table 1. The most prevalent HDs were structural ones in 64% of the patients, followed by rhythm abnormalities in 33%. Among the structural pathologies, the most frequent was congenital heart disease (37.5%), followed by valvular disease (24.8%) and cardiomyopathy (11.3%). HD was known before getting pregnant in 3 out of 4 patients. Despite this, only 15% of them had had a pregestational consultation with the cardiologist, and on average, the gestational age at the time of the first visit to the cardiologist was 19.84 ± 9.13 weeks. During this first evaluation, all of the patients were asymptomatic or had mild cardiovascular symptoms (NYHA functional class I or II). However, more than half of them presented abnormalities in the echocardiographic study, the most frequent findings being moderate to severe valve regurgitation, followed by decreased LVEF and dilation of the systemic ventricle. Regarding the mWHO classification, 16.1% of women with HD in our study had high or very high risk of cardiac complications during pregnancy (mWHO class III or IV).

Table 2 shows the adverse events that occurred during pregnancy in women with HD. With regard to heart complications, one of every five participants presented a MACE (19.9%), and the 17% deterioration of the NYHA functional class. Within the MACEs, the most common were heart failure (11%), arrhythmias (8.6%), and peripartum cardiomyopathy (5%). There was also one thromboembolic event and two cardiac surgeries during pregnancy. Fifteen women (10.6%) were admitted to hospital due to cardiac complications, seven of them to the ICU. There was no aortic dissection, stroke, cardiac arrest or maternal death during the study period. Regarding obstetric adverse events, the most frequent complications were those related to placental insufficiency, such as gestational hypertensive disorders (10%), including a 6.8% frequency of preeclampsia, IUGR (7.2%), and oligohydramnios (7.2%). Five women with HD were admitted due to obstetric complications, mainly because of threatened preterm labor (2.3%) and hypertensive disorders. There was one intrauterine fetal death at 29 weeks related to an umbilical cord knot. It is worth mentioning that 37.1% of women with HD had a cesarean section, the majority of them performed due to obstetric reasons. Prematurity will be analyzed further on as part of the case-control study. Among neonatal complications, the most common was congenital heart disease (13.2%), patent foramen ovale being the most prevalent one, affecting 11 newborns. Twenty newborns required neonatal admission, 4 of them to the NICU. No intraventricular hemorrhage, necrotizing enterocolitis, or retinopathy were detected during the study. Despite cardiac, obstetric, and neonatal complications, 92.4% of women with HD breastfed their newborns.

Table 3 presents the case–control study results, showing the statistical significance, with a special interest in the intrapartum and neonatal outcomes. We found a significantly higher percentage of cesarean sections in the HD group (OR 2.66, 95% CI = 1.66–4.26, *p* < 0.001), mainly due to the higher number of scheduled cesarean sections (22.7% vs. 8.7%, *p* < 0.001), with an OR of 3.08 (95% CI = 1.71–5.56, *p* < 0.001). The most used anesthetic technique during delivery was epidural for both groups; however, we found a 7.8% use of general anesthesia during cesarean section in the case group, much higher than in the control group (OR 10.73, 95% CI = 2.32–49.75, *p* = 0.002). The postpartum hospitalization was, on average, one day longer in the case group, with also more prolonged stays of more than 7 days, OR 2.91 (95% CI = 1.02–8.35, *p* = 0.023). There was no maternal mortality among the study groups.

Regarding the neonatal results, the case group had a significantly higher percentage of male newborns (57% vs. 46%), lower birth weight, with almost twice the control’s rate of low weight under 2.500 gr at birth (OR 1.96 (95% CI 1.09–3.52, *p* = 0.012)). We found also higher prematurity rates under 37 and 34 weeks in women with HD; however, the differences in prematurity rate and the birth weight percentile did not reach statistical significance. The Apgar scores at one minute were significantly lower in the case group than in the control group, without differences in the pH result in umbilical cord blood or in the type of neonatal resuscitation. There was a single neonatal death during the study period, that belonged to the control group, related to extreme prematurity (an urgent cesarean section was performed at 27 weeks due to spontaneous onset of labor and signs of fetal distress). There were no neonatal deaths among the case group.

## 4. Discussion

Women with HD represent 0.27% of our cohort, of whom one-third have congenital heart disease. Despite the fact that all patients have a good basal functional class, up to 22.6% present cardiac adverse events, and the rates of cesarean section, general anesthesia, and prolonged hospitalization are significantly higher compared to controls. With regard to newborns, we find lower weight at birth and lower Apgar test results in women with HD.

The strengths of this work include the use of a hospital cohort of more than 50,000 deliveries, where we found a significant number of pregnant women with HD of different kinds, which allows us to provide data on their characteristics and gestational outcomes by comparing their results with those of controls from the same cohort. Limitations include the retrospective nature of the study, and the heterogeneity within the group of HD patients in terms of etiology and severity, which was not an analysis factor in the comparisons made.

The prevalence of HD in our cohort is 0.27%, similar to the 0.2% described by Lima et al. in the United States, but lower than the 0.7% reported by Ornaghi et al. in Italy, and the 1.82% reported by Fernandez-Campos et al. in Mexico [10,11,12]. These differences could be explained by the fact that the studies ruled by Ornaghi and Fernández-Campos were carried out in reference centers for HD, which could explain the higher prevalence among their patients. In contrast, Lima et al. analyzed the results of more than 8 million deliveries in the United States, from 1000 hospitals, through the data obtained from the National Inpatient Sample (NIS), so that his results are more representative of the general population.

The most prevalent HD among the participants of our study is congenital HD (38%), as is also observed in the majority of studies carried out in industrialized countries. This rate of congenital heart disease is higher than that described by Petrus et al., Hu et al., Ornaghi et al., and Liu et al. in their respective papers, with the lower rate being 25.5% in Liu’s study [1,11,13,14]. In contrast, the studies by Hink et al., Silversides et al., and Fernandez Campos et al. reported a higher proportion of individuals with congenital HD, which was present in up to two-thirds of the participants in the Fernandez-Campos’s study [12,15,16]. As these authors noted, the presence of congenital HD among pregnant women is increasing due to the therapeutic advances that have prolonged their survival and quality of life [15]. In addition, among different types of HD, women with congenital HD tend to have good gestational outcomes, as concluded by Lima as follows: “the highest percentages of MACEs occurred in women with cardiomyopathy and the lowest in congenital heart diseases (44% vs. 6.2%)” [10].

Although the proportions of the different types of HD vary among studies, stratifying the risk of cardiovascular complications during pregnancy using the mWHO classification allows us to gain a sense of their severity. In this study, 16% of the participants with HD have a high or very high risk of cardiac adverse events (class III or IV of the WHO m classification), similar to that described in some of the previously cited studies [12,16,17] and close to the final percentage of MACEs that occurred in our study, which was 19.9%. In the most recent literature, the percentage of MACEs or serious cardiac complications ranges between 10.7% and 23.6% [1,10,11,12,13,16,17,18,19], with an average of approximately 16% in studies with larger sample sizes [10,16,17,18,19]. Again, these differences between the studies depend on the types of HD included and their a priori risk of complications. According to Roos-Hesselink et al., the number of high-risk pregnancies (mWHO class IV) increased from 0.7% between 2007 and 2010 to 10.9% between 2015 and 2018, although the vast majority of the patients included in their international study had a good functional class (94% class I–II NYHA), as it occurs in the present work [17]. There are no maternal deaths observed in this study, similarly, in the literature, mortality associated with pregnancy in women with HD is usually less than or equal to 2% according to studies published since 2015 [1,13,14,15,16,17,20,21]. In the words of Silversides: “pregnancy in women with heart disease continues to be associated with significant morbidity, although mortality is rare” [16].

Regarding delivery outcomes, among our women with HD, the cesarean section rate is 37.1%, twice that among controls and similar to that described by Ramage et al. (2019) and Lammers et al. (2021) in women with congenital HD [22,23]. The rates of cesarean section in women with HD vary widely in the literature, depending on socioeconomic and temporal factors and the characteristics of the study population. Overall, there seems to be a downward trend, probably related to the growing consensus to prioritize vaginal delivery in this group of patients if the maternal and fetal situation allows it. Regarding the reason for cesarean deliveries, obstetrical motives are more frequent than cardiological ones, as is the case in our study [14,15]. On the other hand, we find a significantly higher rate of general anesthesia use (7.8% vs. 0.8%, *p* < 0.01), similar to the findings of Hu et al. (6.72%) and Hink et al. (10%) [13,15]. The reasons for using general anesthesia include cardiopulmonary decompensation, current anticoagulation, severe thrombocytopenia, and maternal refusal of neuraxial anesthesia [24].

Regarding the neonatal outcomes, mean gestational age in our study is 38 weeks for both groups, but the average birth weight is 134 g lower in the HD group, similar to the results described by Hrycyk et al. and Lammers et al. [23,25]. The incidence of congenital HD in the case group’s neonates is 13.2%, which is higher than that shown by Petrus et al. (11.7%) or Hink et al. (9.2%) and lower than that described by Lammers et al. (17.7%) [1,15,23]. These differences are probably due to different percentages of mothers with congenital HD within studies, as this is the main risk factor. There is one intrauterine fetal death in the case group (0.7%) and none in the control group; however, in the neonatal period, there is one death in the control group (0.4%) but none in the case group. These results are consistent with those obtained in other studies with larger sample sizes that report an intrauterine mortality rate that ranges from 0.3% to 2.2% and a neonatal mortality rate of 0.6–0.7% in women with HD [14,17,21].

## 5. Conclusions

The prevalence of HD in pregnant women is increasing, especially due to the good survival of patients with congenital HD. This study shows that HD increases maternal morbidity during pregnancy and it is associated with higher rates of cesarean section and low birth weight in neonates compared to healthy controls. However, the majority of them do not request a pregestational consultation for cardiological assessment. We aim to provide information on the obstetric outcomes of women with HD to help clinicians in the decision-making process, highlighting the importance of a multidisciplinary and individualized management during pregnancy and delivery.

## Figures and Tables

**Table 1:** Maternal characteristics of women with heart disease.

	*n* = 141
Personal history	
Smoking, *n*, %	13 (9.2%)
Diabetes mellitus, *n*, %	3 (2.1%)
Chronic hypertension, *n*, %	5 (3.5%)
Stroke, *n*, %	2 (1.4%)
Heart failure, *n*, %	11 (7.8%)
Cardiovascular surgeries, *n*, %	36 (25.5%)
Pre-pregnancy cardiovascular medication	34 (24.1%)
Known heart disease, *n*, %	105 (74.5%)
In vitro fertilization, *n*, %	13 (9.2%)
Basal cardiological characteristics	
Pre-pregnancy consultation with cardiologist, *n*, %	21 (14.8%)
Type of heart disease, *n*, %:	
Congenital heart disease	53 (37.5%)
Cardiomyopathy	16 (11.3%)
Ischemic heart disease	2 (1.4%)
Marfan syndrome	2 (1.4%)
Valve disease	35 (24.8%)
Rheumatic valve disease	9 (6.3%)
Valve prosthesis	6 (4.3%)
Arrhythmia	46 (32.6%)
Arrhythmia device	4 (2.9%)
Basal echocardiographic parameters, *n*, %:	
Systemic ventricle ejection dysfunction (SVEF) < 55%	13 (9.2%)
Systemic ventricle dilatation	13 (9.2%)
Sub-pulmonary ventricle dysfunction	10 (7.09%)
Ascending aortic dilatation	6 (4.4%)
Systemic ventricle outflow tract obstruction	7 (5.0%)
Pulmonary hypertension	2 (1.4%)
Valvular insufficiency (moderate or severe)	22 (15.6%)
New York Heart Association (NYHA) functional classification, *n*, %:	
I	123 (87.2%)
II	18 (12.8%)
Modified World Health Organization (mWHO) classification, *n*, %:	
I	34 (24.1%)
II	48 (34.0%)
II–III	36 (25.5%)
III	20 (14.2%)
IV	3 (2.1%)

**Table 2:** Adverse events during pregnancy in women with heart disease.

	*n* = 141
Use of CV medication during current pregnancy *n*, %	47 (33.3%)
Cardiovascular complications	32/141 (22.6%)
Deterioration of NYHA functional class, *n*, %	24 (17.0%)
MACEs (major maternal CV events), *n*, %	28/141 (19.9%)
Heart failure, *n*, %	15 (10.6%)
Peripartum cardiomyopathy, *n*, %	7 (4.9%)
Acute pulmonary oedema, *n*, %	5 (3.6%)
Thromboembolic complication, *n*, %	1 (0.7%)
Arrhythmic complication, *n*, %	12 (8.6%)
Cardiac surgery during pregnancy, *n*, %	2 (1.6%)
Hospital admission due to cardiovascular complication, *n*, %	15 (10.6%)
Maternal admission to the intensive care unit (ICU), *n*, %	7 (5.0%)
Duration of ICU admission (days) (*n* = 7), mean ± SD	7.7 ± 7.1
Maternal Death, n, %	0 (0%)
	*n* = 132
Obstetric complications	32/132 (24.2%)
Stillbirth, *n*, %	1 (0.7%)
Threatened preterm labor, *n*, %	3 (2.3%)
Gestational hypertensive disorder, *n*, %	13 (9.8%)
Preeclampsia, *n*, %	9 (6.8%)
Fetal macrosomia *n*, %	2 (1.6%)
Oligohydramnios, *n*, %	9 (7.2%)
Intrauterine growth restriction (IUGR), *n*, %	9 (7.9%)
Hospital admission for obstetric reasons (other than delivery), *n*, %	5 (3.8%)
Postpartum hemorrhage, *n*, %	4 (3.2%)
Other obstetric variables	
Cardiovascular cause for C-section, *n*, %	7 (14.6%)
Obstetric cause for C-section, *n*, %	36 (75%)
Both CV and obstetric cause for C-section, *n*, %	5 (10.4%)
No breastfeeding, *n*, %	10 (7.6%)
	*n* = 136
Neonatal complications	38/136 (27.9%)
Congenital heart disease, *n*, %	18 (13.2%)
Neonatal admission, *n*, %	20 (14.7%)
Duration of neonatal admission (days) (*n* = 20), mean ± SD	6.5 ± 7.0
Neonatal intensive care unit admission, *n*, %	4 (3.1%)
Duration of neonatal intensive care unit admission (*n* = 4), mean ± SD	5 ± 3.27

**Table 3:** Comparative analysis of delivery and neonatal outcomes between women with heart disease (cases) and women without heart disease (controls).

	Total	Cases	Controls	*p* Value
	*n* = 396	*n* = 132	*n* = 264	
Maternal Characteristics				
Nulliparity, *n*, %	223 (56.3%)	73 (55.3%)	150 (56.8%)	0.8 ^2^
Twin pregnancy, *n*, %	12 (3.0%)	4 (3.0%)	8 (3.0%)	>0.9 ^2^
Maternal age (years), mean, SD	32.71 ± 5.84	33.17 ± 5.51	32.49 ± 5.99	0.3 ^2^
Maternal age < 20 years	9 (2.3%)	2 (1.5%)	7 (2.7%)	0.5 ^2^
Maternal age 20–35 years	252 (63.6%)	82 (62.1%)	170 (64.3%)	0.7 ^2^
Maternal age 36–39 years	98 (24.7%)	33 (25.0%)	65 (24.6%)	>0.9 ^2^
Maternal age > 40 years	37 (9.3%)	15 (11.3%)	22 (8.3%)	0.3 ^2^
Delivery results				
Onset of labor, *n*, %:				
Spontaneous	242 (61.1%)	60 (45.5%)	182 (68.9%)	<0.001 ^2^
Induction	100 (25.3%)	41 (31.1%)	59 (22.3%)	0.057 ^2^
Type of anesthesia, *n*, %:				
None	26 (6.6%)	6 (4.7%)	20 (7.6%)	0.3 ^2^
Local	21 (5.4%)	3 (2.3%)	18 (6.8%)	0.048 ^2^
Rachidial	45 (11.4%)	23 (17.4%)	22 (8.3%)	0.006 ^2^
Epidural	288 (72.7%)	86 (65.1%)	202 (76.5%)	0.052 ^2^
General	12 (3.1%)	10 (7.8%)	2 (0.8%)	<0.001 ^2^
Type of delivery, *n*, %:				
Total C-sections	97 (24.5%)	49 (37.1%)	48 (18.2%)	<0.001 ^2^
Unplanned C-sections	44 (11.1%)	19 (14.4%)	25 (9.5%)	0.917
Scheduled C-section	53 (13.4%)	30 (22.7%)	23 (8.7%)	<0.001 ^2^
Total vaginal deliveries	298 (75.3%)	82 (62.1%)	216 (81.8%)	<0.001 ^2^
Eutocic vaginal delivery	234 (59.1%)	59 (44.7%)	175 (66.3%)	<0.001 ^2^
Assisted vaginal delivery	64 (16.2%)	23 (17.4%)	41 (15.5%)	0.6 ^2^
Postpartum maternal admission (days), mean ± SD	2.81 ± 2.44	3.49 ± 2.67	2.50 ± 2.26	<0.001 ^2^
Postpartum maternal admission ≥ 7 days, *n*, %	15 (3.9%)	9 (7.2%)	6 (2.3%)	0.025 ^2^
Neonatal results	*n* = 408	*n* = 136	*n* = 272	
Male, *n*, %	201 (49.3%)	76 (55.9%)	125 (46.0%)	0.034 ^2^
Gestational age (GA), mean ± SD	38.52 ± 2.32	38.23 ± 2.10	38.66 ± 2.41	0.095 ^2^
GA < 37 weeks	41 (10.0%)	18 (13.2%)	23 (8.5%)	0.14 ^2^
GA < 34 weeks	13 (3.2%)	5 (3.6%)	8 (2.9%)	0.7 ^2^
GA < 32 weeks	7 (1.7%)	1 (0.7%)	6 (2.2%)	0.2 ^2^
Weight (grams), mean ± SD	3101.87 ± 600.84	3012.40 ± 595.95	3146.77 ± 599.35	0.035 ^2^
Weight < 2.500 g, *n*, %	53 (13.0%)	25 (18.3%)	28 (10.3%)	0.026 ^2^
Weight percentile, *n*, %	50.57 ± 31.54	46.62 ± 31.54	52.51 ± 31.42	0.078 ^2^
Weight < 10th percentile, *n*, %	48 (11.8%)	20 (14.7%)	28 (10.3%)	0.2 ^2^
Neonatal reanimation, *n*, %				
None	315 (77.2%)	99 (72.8%)	216 (79.4%)	0.6 ^2^
Aspiration of nasopharyngeal secretions	14 (3.4%)	4 (2.9%)	10 (3.7%)	0.8 ^2^
Oxygen therapy	15 (3.7%)	4 (2.9%)	11 (4.0%)	0.6 ^2^
Positive pressure ventilation	32 (7.8%)	13 (9.6%)	19 (6.9%)	0.3 ^2^
Invasive ventilation	8 (1.9%)	2 (1.5%)	6 (2.2%)	0.7 ^2^
Apgar test 1st minute, mean ± SD	8.54 ± 1.27	8.35 ± 1.45	8.63 ± 1.17	0.043 ^2^
Apgar test 1st minute < 7, *n*, %	28 (6.9%)	12 (9.0%)	16 (5.9%)	0.3 ^2^
Apgar test 5th minute, mean ± SD	9.51 ± 1.00	9.37 ± 1.20	9.58 ± 0.88	0.05 ^2^
Apgar test 5th minute < 7, *n*, %	4 (1.0%)	2 (1.5%)	2 (0.7%)	0.5 ^2^
Arterial Ph umbilical cord, mean ± SD	7.28 ± 0.08	7.28 ± 0.08	7.28 ± 0.08	0.8 ^2^
Arterial Ph umbilical cord < 7, *n*, %	3 (0.8%)	0 (0%)	3 (1.1%)	0.14 ^2^
Neonatal death, *n*, %	1 (0.2%)	0 (0%)	1 (0.4%)	0.4 ^2^

^2^ Random intercept logistic regression.

## Data Availability

The data used to support the findings of the present study are available from the corresponding author upon request.
